# Comparative models disentangle drivers of fruit production variability of an economically and ecologically important long-lived Amazonian tree

**DOI:** 10.1038/s41598-021-81948-4

**Published:** 2021-01-28

**Authors:** Christina L. Staudhammer, Lúcia Helena O. Wadt, Karen A. Kainer, Thiago Augusto da Cunha

**Affiliations:** 1 Department of Biological Sciences, University of Alabama, P.O. Box 870344, Tuscaloosa, AL 35487 USA; 2 Centro de Pesquisa Agroflorestal de Rondônia (Embrapa Rondônia), BR 364, km 5,5, Caixa Postal 127, Porto Velho, Rondônia CEP 76815-800 Brazil; 3 School of Forest Resources and Conservation, and Center for Latin American Studies, University of Florida, P.O. Box 110410, Gainesville, FL 32611-0410 USA; 4 Universidade Federal do Acre, Rodovia BR 364, Km 04, Distrito Industrial, Rio Branco, Acre Brazil

**Keywords:** Forest ecology, Tropical ecology

## Abstract

Trees in the upper canopy contribute disproportionately to forest ecosystem productivity. The large, canopy-emergent *Bertholletia excelsa* also supports a multimillion-dollar commodity crop (Brazil nut), harvested almost exclusively from Amazonian forests. *B. excelsa* fruit production, however is extremely variable within populations and years, destabilizing local harvester livelihoods and the extractive economy. To understand this variability, data were collected in Acre, Brazil over 10 years at two sites with similar climate and forest types, but different fruit production levels, despite their proximity (~ 30 km). One site consistently produced more fruit, showed less individual- and population-level variability, and had significantly higher soil P and K levels. The strongest predictor of fruit production was crown area. Elevation and sapwood area also significantly impacted fruit production, but effects differed by site. While number of wet days and dry season vapor pressure prior to flowering were significant production predictors, no climatic variables completely captured annual observed variation. Trees on the site with higher available P and K produced nearly three times more fruits, and appeared more resilient to prolonged drought and drier atmospheric conditions. Management activities, such as targeted fertilization, may shield income-dependent harvesters from expected climate changes and production swings, ultimately contributing to conservation of old growth forests where this species thrives.

## Introduction

The influence of canopy emergent trees on tropical forest structure and function is immense. They support wildlife, serve as physical substrates for other biota, shape microclimates, and contribute disproportionately to forest carbon stocks^1^. In basin-wide analyses of hyperdominance in unflooded Amazonian forests, large trees contributed disproportionately larger amounts to total biomass and productivity^2^. Their belowground influence on water and nutrient cycling may be just as critical. While understory trees can have deep roots, tall trees preferentially tap water from layers below one meter depth^3^, suggesting that canopy trees also play a disproportional role in accessing the deep soil water that mitigates seasonal Amazonian droughts^4^.

*Bertholletia excelsa* is one of the top 20 most dominant Amazonian species in terms of forest carbon storage and productivity^2^ and can live for centuries^5^. It represents 1.31% of the total aboveground biomass of the unflooded Amazonian forests, ranking 3rd and 4th of all species in terms of aboveground biomass and woody productivity, respectively^2^. Radio-carbon dating of *B. excelsa* individuals suggests lifespans > 1000 years^5^, and reproductive output is estimated to initiate in the first two centuries^6^, and last until individuals near senescence. But *B. excelsa*’s critical ecological role is only the tip of the iceberg, because this canopy-emergent tree also sustains a multimillion-dollar commodity crop known as Brazil nuts. Harvested almost solely from mature tropical forests, the 10–25 nuts (botanically seeds) remain inside a globose woody fruit that falls to the ground where it remains until extraction, almost exclusively by agoutis (*Dasyprocta* sp.) or humans. This single species supports food security, cultural heritage, and livelihoods of thousands of Amazonian residents, triggering conservation of standing Brazil nut-rich forests in Brazil, Bolivia and Peru^7^. At current harvest intensities, the longstanding collection of *B. excelsa* seeds does not seem to compromise future generations, as suggested by most research^6,8,9^ (but see^10^). Notwithstanding, *B. excelsa* fruit production varies by year and fluctuates greatly in individual trees^11,12^, leading to research focused on variability and suggesting that silvicultural interventions and best management practices (e.g., liana cutting^13^) could potentially increase productivity. In recent years, interest in the causes and mechanisms of variable fruit production and options for its control have been heightened by alterations in nut market prices^14^, intensive deforestation in the lower Amazon basin, and growing understanding of the extent to which this valuable resource remains untapped in other parts of Brazil nut-rich Amazonia^15^.

We aim to understand what drives fruit production variation of canopy-emergent trees by comparing two *B. excelsa* populations, Filipinas and Cachoeira, in the Acre River Valley of the western Brazilian Amazon. While both sites are located in sustainable use reserves and are only ~ 30 km apart, Cachoeira has historically produced three times as many fruits as Filipinas. By comparing these sites over a 10-year period and supplementing annual tree and fruit production data with soil and climate data, we partitioned variability into site-specific, tree-specific and climate-induced effects. We hypothesized that fruit production at these sites would show similar patterns over time, and common driving variables. Using a generalized linear mixed model, we accounted for the non-normal nature of the production response with a generalized Poisson distribution in a repeated measures framework. Elucidation of driving factors that control seed production can answer questions about the fate of long-lived species. For *B. excelsa* in particular, such knowledge could provide greater stability for communities of harvesters and the Brazil nut extractive economy.

## Results

**Figure 1:**
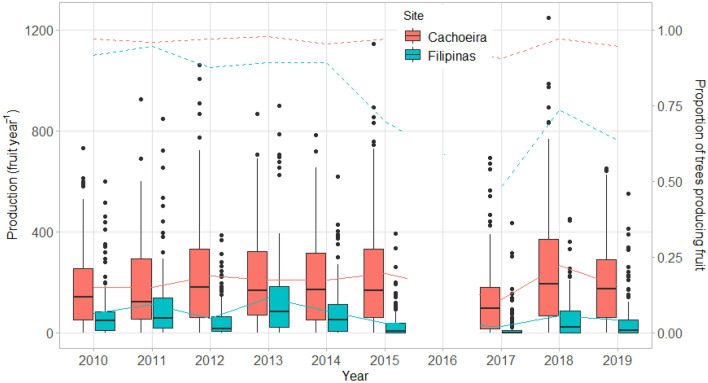
Distribution of annual production by site and year. Boxes outline interquartile range, vertical lines extend to 1.5 × interquartile range, with outliers individually marked. Solid and dashed lines join annual mean and proportion of trees producing fruit by site, respectively. In each site, n = 129 trees were observed per year except in 2011, when 124 trees were observed in Cachoeira and 128 in Filipinas, and in 2017–2018, when 128 trees were observed in Cachoeira.

At site level, there were large differences in average production and production variability (Fig. 1). While both sites had trees that produced large fruit numbers (> 850 fruit year^−1^), Cachoeira’s trees produced on average nearly three times as many fruits as those in Filipinas over the 10-year period. The distribution of production by tree was very skewed, especially in Filipinas, with the median far lower than the mean in all years. Filipinas experienced its highest production and lowest variability in 2013, whereas Cachoeira’s highest production year was 2018 and variability was fairly constant over time.

We found that population variability (*CVp*) and individual variation in number of fruits (*CVi*) were higher in Filipinas (0.497 and 1.162, respectively) compared to Cachoeira (0.192 and 0.670). Overall synchrony of fruiting in Cachoeira and Filipinas was minimal (*S* = 0.068 and 0.223, respectively), confirming that this species does not exhibit masting behavior^11^. While patterns of productivity revealed that 12% and 5% of trees followed a strict pattern of alternating years of high and low production at Cachoeira and Filipinas, respectively, 70% of trees had this pattern in 5 of the possible 7 annual transitions in both sites. On average, tree-level AR(1) coefficients showed a weak high-low alternating pattern in Cachoeira (− 0.17) and no pattern in Filipinas (− 0.06).

### Climate and soils

**Figure 2:**
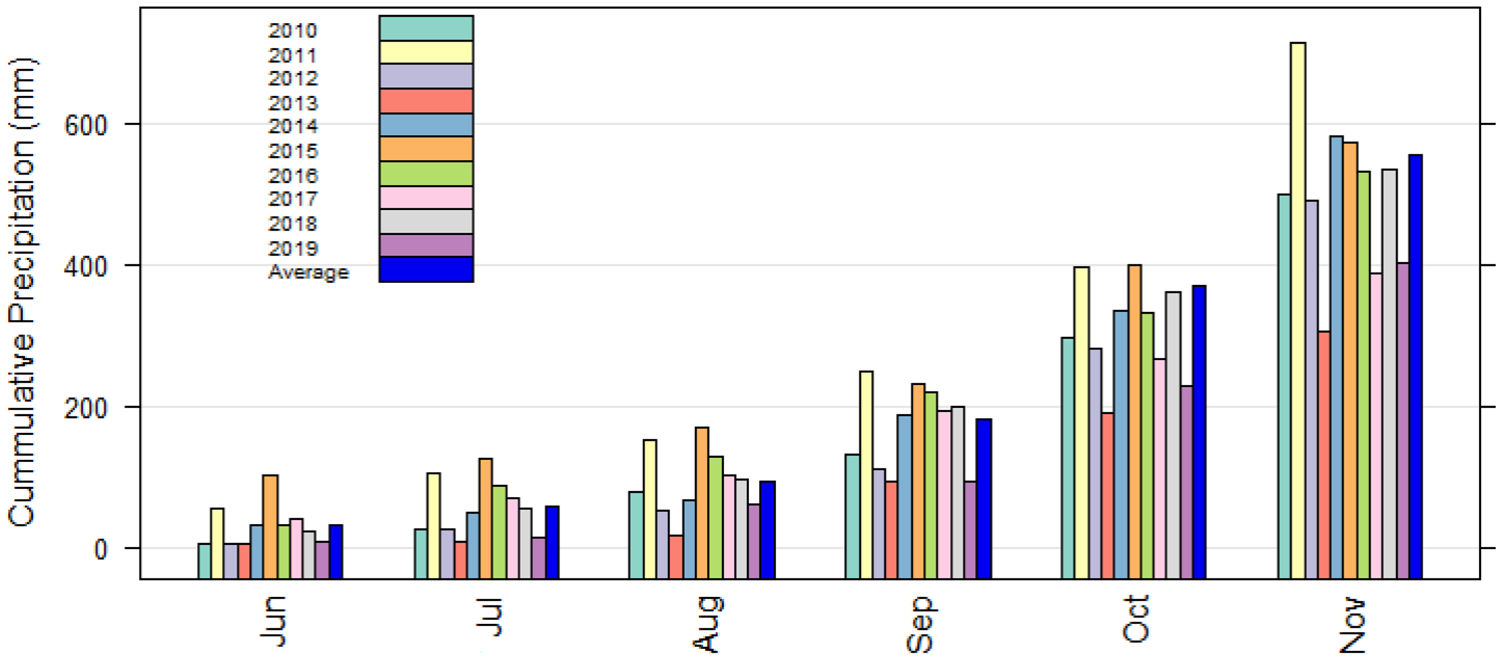
Cumulative precipitation from dry season prior to flowering through flowering, by production year, June–November, 2008 through 2017, corresponding to 2010–2019 production years.

During the study, rainfall was variable, with large differences in cumulative precipitation during the dry-season and flowering period (Fig. 2). In particular, precipitation during the dry season prior to flowering (DPF: June–August) corresponding to production years 2012–2014 and 2019 was much lower than the long-term average (1901–2019), while precipitation during DPF of production year 2011 was much higher. Cumulative precipitation of 2011 production year continued to be very high for the dry season through flowering (DTF: June–November) period, whereas DTF precipitation in production years 2013, 2017, and 2019 was very low.

Spearman correlation analysis revealed that few climate variables had high correlations with fruit production, especially in Filipinas. While no correlation met the *p* < 0.05 (|r|≥ 0.68) threshold of significance, increased minimum (nighttime) temperatures during both DPF and DTF in Cachoeira were associated with lower production values (r = −0.4), and higher precipitation was associated with lower production during DPF in Filipinas (r = −0.37).

Few soil characteristics differed by site (Table 1). Only phosphorous and potassium were significantly different by site (*p* < 0.05), with higher values in Cachoeira. While Cachoeira’s sodium and calcium were > 60% higher, and iron ~ 50% lower, these differences were not significant.

**Table 1:** Average soil characteristics by site, and results of F-test for location effects.

Variable	Cachoeira	Filipinas	Pr > F_(1,2)_
pH (H_2_O)	4.41	4.17	0.222
Organic material (g kg^−1^)	12.57	13.38	0.756
N (g kg^−1^)	0.96	0.78	0.339
P (mg dm^−3^)	5.16	3.83	< 0.0001
K (mg dm^−3^)	55.48	35.03	0.034
Na (mg dm^−3^)	1.97	1.21	0.225
Ca (cmolc dm^−3^)	0.17	0.09	0.290
Mg (cmolc dm^−3^)	0.33	0.19	0.060
H Al (cmolc dm^−3^)	4.14	3.85	0.711
t (cmolc dm^−3^)	2.73	2.4	0.521
Fe (mg dm^−3^)	127.4	241.3	0.214
Mn (mg dm^−3^)	41.98	35.75	0.783
Cu (mg dm^−3^)	0.86	0.86	0.995
Clay content (g kg^−1^)	173.9	133.2	0.467
Particulate density (mg m^−3^)	2.58	2.65	0.444
Porosity (%)	47.86	50.36	0.477

### Models of fruit production

**Figure 3:**
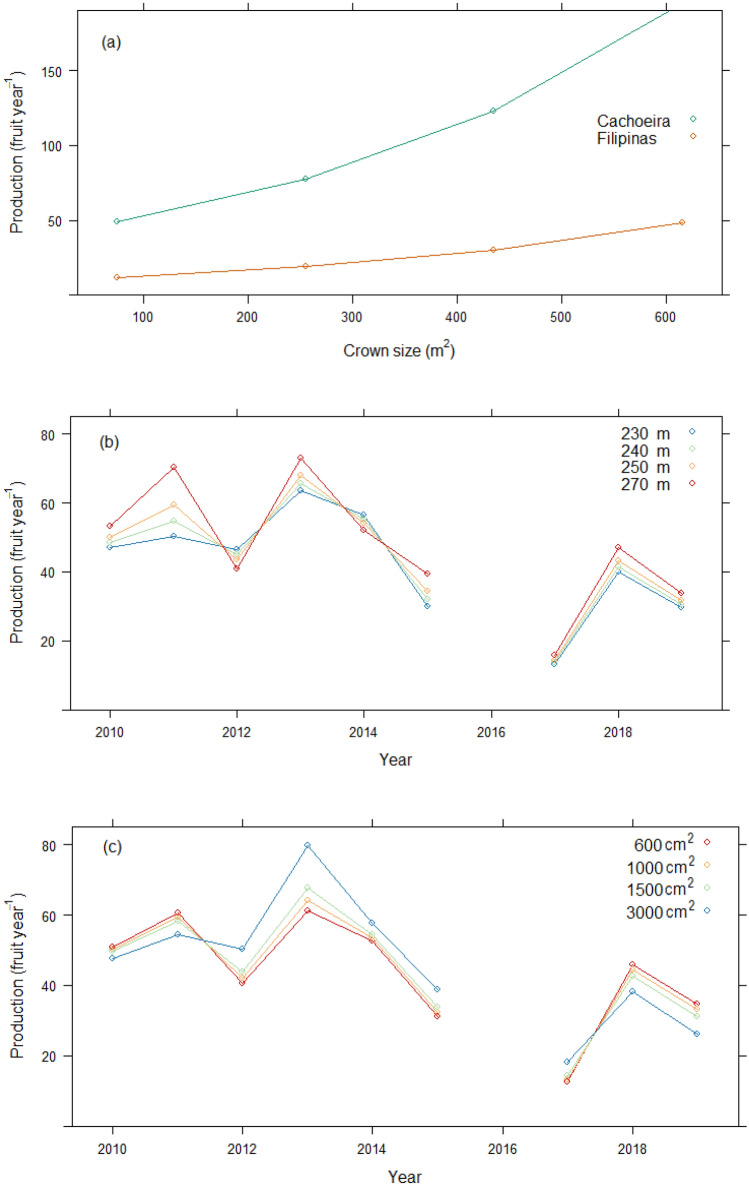
Model 1 marginal mean values of annual fruit production: (**a**) by site and crown size (m^2^), (**b**) by year and four values of elevation (m), and (**c**) by year and sapwood area (cm^2^).

When considering all trees in both sites (*Model 1*), production was significantly higher with larger crowns and better crown form (Table 2). Crown size was a better predictor of production than tree diameter and was the most important predictor after year and site (Fig. 3a). The effect of competition from neighboring trees was not a significant predictor. While elevation was significant, in agreement with Thomas et al.^16^, its effect was only evident in 2011, when the highest elevation trees produced more than the lowest (Fig. 3b). Similarly, while sapwood area also was associated with production, it was only significant in 2013, when trees with > 3000 cm^2^ sapwood area produced significantly more than those with < 1000 cm^2^ (Fig. 3c).

**Table 2:** F-values and statistical significance from models of annual production, using (1) year as the indicator of annual conditions, and (2) climate variables instead of year, (3a) small trees only (< 100 cm DBH), (3b) large trees only (≥ 100 cm), (4a) large trees in Cachoeira, and (4b) large trees in Filipinas. Models 3–5 use year as the indicator of annual conditions (Full model statistics available in SI Table S1).

Effect	Model 1 Year	Model 2 Climate	Model 3a Small trees	Model 3b Large trees	Model 4a Cachoeira large trees	Model 4b Filipinas large trees
Year	64.26****		22.07****	36.46****	10.18****	40.32****
Site	104.84****	115.47****	104.97****	36.13****		
Year × site	39.32****		16.98****	16.34****		
Crown size	26.53****	26.84****	19.49****	15.98****	6.02*	8.83**
Crown form	8.62***	8.6***	6.69**			
Elevation	0.64		0.34	4.08*		1.51
Elevation × year	2.25*		3.07**			
Elevation × site			0.14			
Elevation × year × site			2.36*			
Sapwood area	0.05	0.01	3.14	0.00	0.11	0.02
Sapwood area × year	3.57***			1.77	4.8****	2.05*
Sapwood area × site				0.00		
Sapwood area × year × site				2.9**		
VAP (DTF)		248.6****				
VAP × site		25.12****				
VAP × sapwood area		5.03*				
Wet days (DPF)		30.91****				
BA growth			13.71***			
BA growth × year			2.36*			
BA growth × site			7.78**			

*VAP* vapor pressure, *DTF* dry season through fruiting, *DPF* dry season prior to fruiting, *BA* basal area.

*p < 0.05; **p < 0.01; ***p < 0.001; ****p < 0.0001.

**Figure 4:**
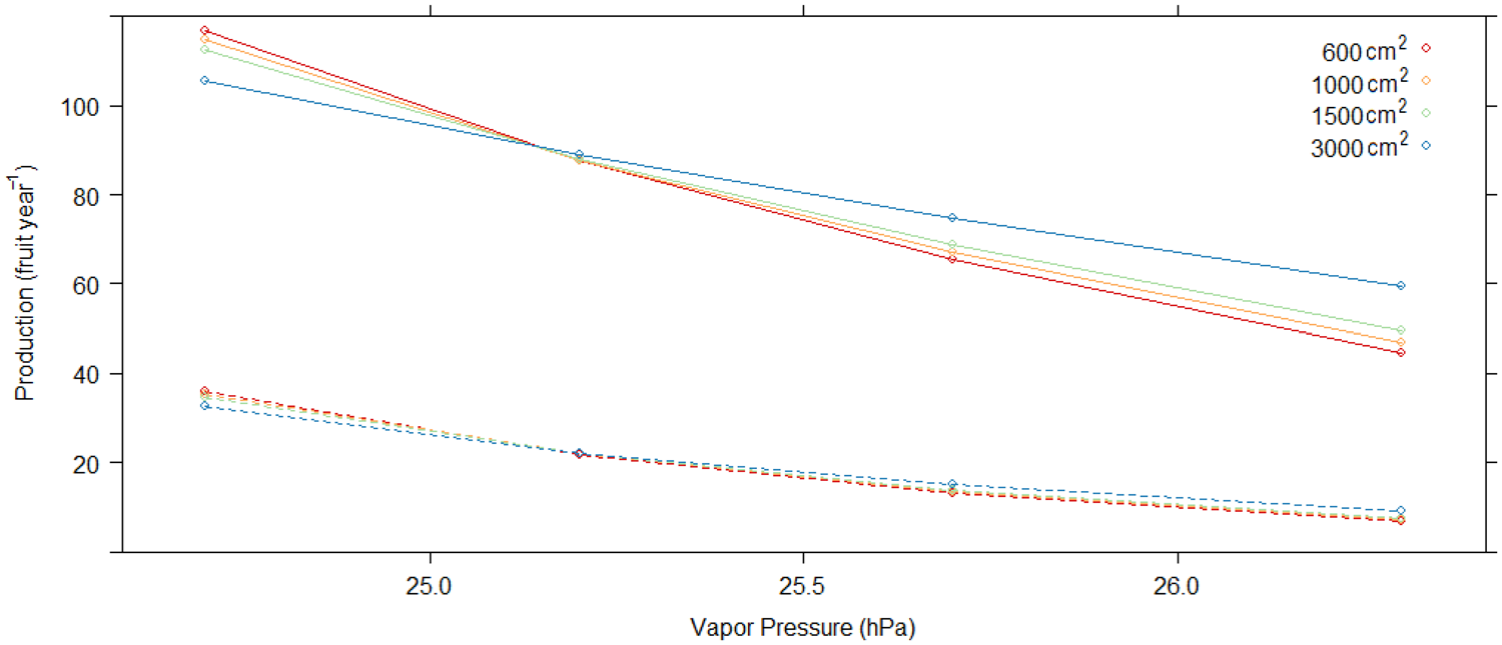
Model 2 marginal mean values of annual fruit production by vapor pressure (hPa) during dry season through flowering (DTF) at four values of sapwood area (cm^2^). Solid lines = Cachoeira, dashed lines = Filipinas.

When using climate variables instead of year as a predictor (*Model 2*), site and crown size and form continued to be highly significant predictors of fruit production, but elevation was not (Table 2). The best fit model included vapor pressure (VAP) during DTF and number of wet days during DPF. However, the AIC indicated far less support for this model versus that of Model 1 (with year). Fruit production was marginally higher as number of wet days during DPF increased, and generally lower as VAP increased. Like the year variable, VAP interacted with sapwood area and site in predicting production. In Cachoeira, the effect of VAP was more pronounced, especially in trees with smaller sapwood areas (Fig. 4). In Filipinas, this effect was dampened.

**Figure 5:**
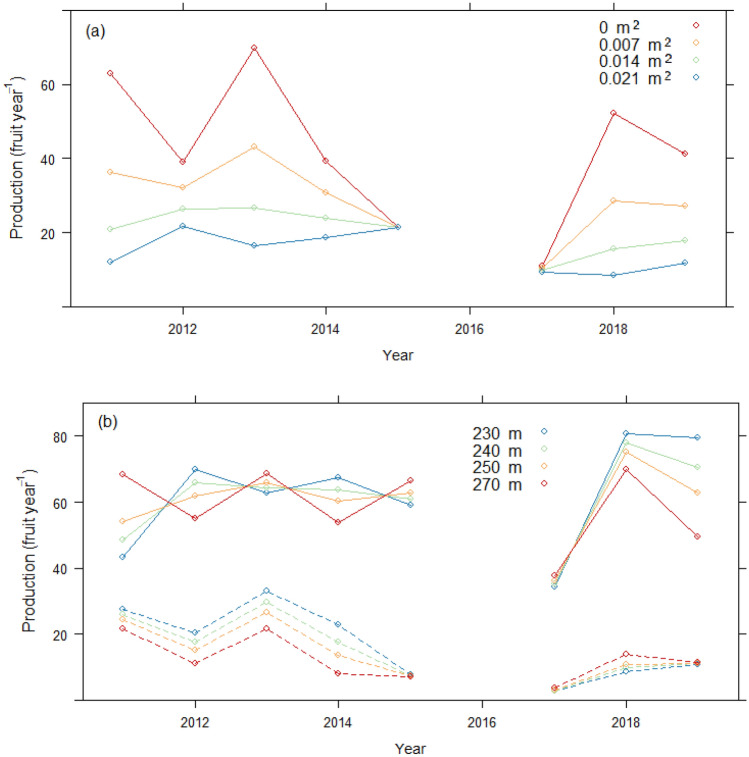
Model 3a marginal mean values of annual fruit production of small trees (DBH< 100 cm): by four values of (**a**) basal area growth (m^2^ year^−1^), and (**b**) elevation (m). Solid lines = Cachoeira, dashed lines = Filipinas.

Because previous work has shown that *B. excelsa* growth and reproduction trade-offs seem to exist only in small trees and not larger trees that have structural potential to maximize production^17^, we disentangled tree size in Models 3a and b, respectively. Similar to Model 1, year, site, and crown size were the strongest predictors in models of both small (those with diameter at breast height [DBH] < 100 cm, measured at 1.3 m above ground level and large (DBH ≥ 100 cm) tree production. Basal area growth in the year previous to fruit production was also a very strong predictor, but as expected, only for smaller trees. Significantly higher production in smaller trees in 2011, 2013, 2018 and 2019 was observed in those trees that were growing more slowly. This apparent and expected small-tree tradeoff between production and growth was weaker in all other years, especially in the driest (2015, 2017), when production seemed completely independent of growth (Fig. 5a). In Filipinas, smaller trees growing at higher elevations had significantly lower production during 2012–2014, whereas differences among elevations in other years were not significant (Fig. 5b). Similarly, Cachoeira’s smaller trees growing at higher elevations also tended to demonstrate lower production, but only significantly so in 2019. In contrast, in 2011, Cachoeira’s small-tree production was higher for high elevation trees, but was only significant for those growing at extreme ends of the elevation distribution.

**Figure 6:**
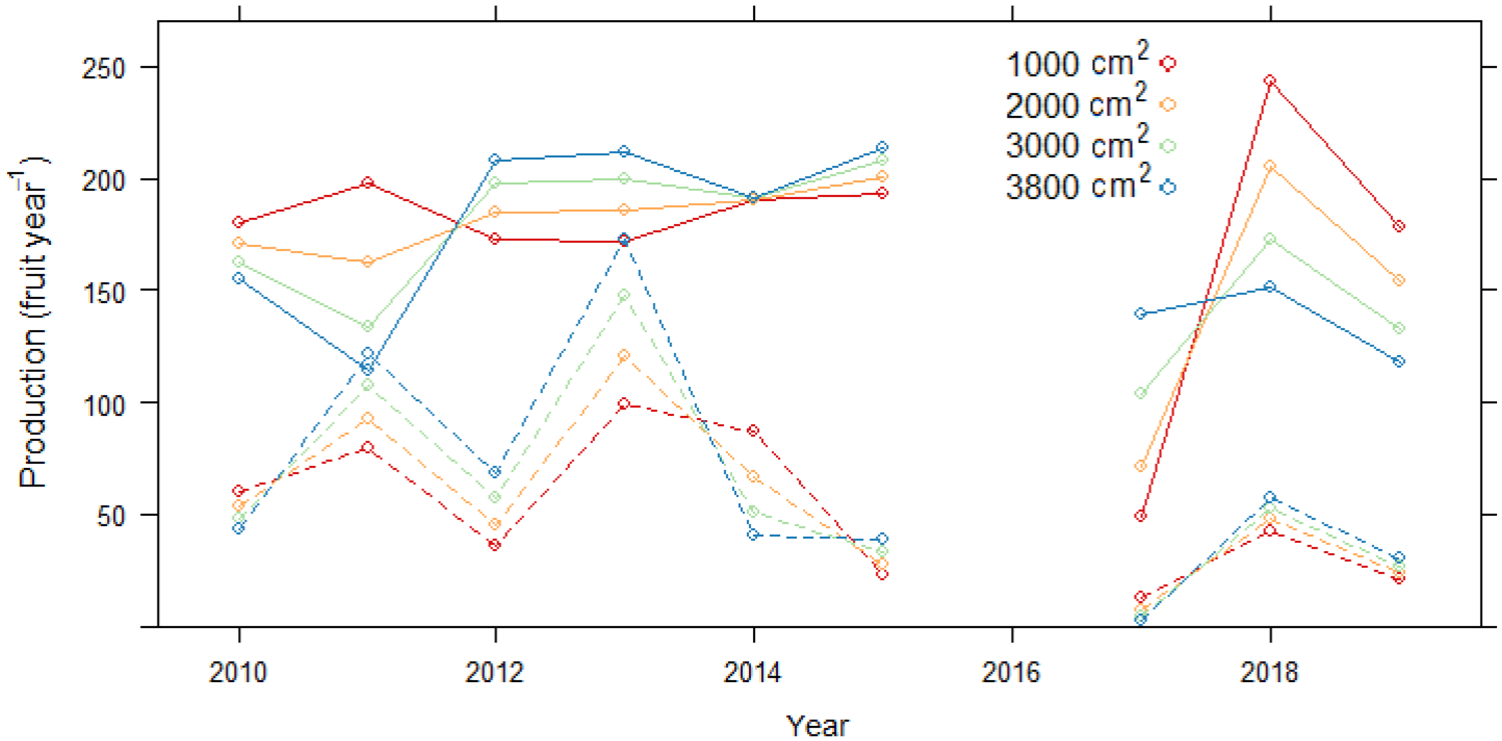
Model 3b marginal mean values of annual fruit production of large trees (DBH ≥ 100 cm) by four values of sapwood area (cm^2^). Solid lines = Cachoeira, dashed lines = Filipinas.

Production in large trees (Model 3b) was more than twice as high as in smaller trees. Again year, site, and crown size were the strongest predictors (Table 2). There was a weakly positive effect of elevation on fruit production. Like Model 1, production was significantly different by sapwood area, but this effect depended on year and site (Fig. 6). During 2017, Cachoeira’s large trees produced significantly more fruit when sapwood areas were larger; however, the opposite was true during 2011 and 2018 (Fig. 6). High uncertainty in Filipinas effected no significant differences by sapwood values for any year.

When focusing solely on large trees by site, the best fitting models (*Models 4a* and *b*) were much simpler, with very few significant effects (Table 2). Crown size was the most important predictor, after year, for both sites. Whereas elevation was not a significant predictor in either site, its inclusion in the Filipinas model led to a lower (better) AIC statistic. For both sites, sapwood area had a significant association with fruit production, which varied by year. Similar to Model 3b, in 2017, Cachoeira's large trees with larger sapwood areas produced significantly more than those with smaller sapwood areas, whereas in all other years, sapwood was not significantly associated with production. The only significant differences in Filipinas occurred in 2013, when higher production values were associated with larger sapwood areas (similar to Fig. 6).

## Discussion

We set out to disentangle the manifold and interacting drivers of fruit production of large, long-lived tropical canopy trees. We used two *B. excelsa* populations as models given the critical importance of this single species to ecosystem processes, Amazonian livelihoods, and tropical biodiversity conservation. Our findings uncovered that over 10 years, one site (Cachoeira) consistently generated production levels that were threefold higher than that of the other site (Filipinas). Fruit production variation at Cachoeira was also relatively constant at both individual and population levels compared to Filipinas. Yet as anticipated in the tropics (versus temperate regions) where low climate variability minimizes resource variation^18^, neither population exhibited masting behavior as indicated by synchrony (*S*).

Given that we hypothesized that fruit production would show similar patterns over time, and common driving variables, we expected weather and weather cues to play important roles in fruit production. Because our research sites are only ~ 30 km apart, we assumed that each population and individual tree experienced approximately the same weather and climatic cues. Our climate model indicated that more wet days during the narrow 3-month dry season prior to flowering resulted in increased fruit production. Furthermore, the model also indicated that when wetter atmospheric conditions (represented by VAP) were present and extended beyond the dry season into the flowering period, fruit production tended to be reduced. Still, models that used the simple “year” variable to explain fruit production variation (versus multiple specific, albeit remote climate variables) had better statistical fit. This leads us to question what overall weather conditions might have caused the extremely low and highly variable production levels of 2017; in Filipinas, more than half of the trees did not produce any fruits (Fig. 1). Local Brazil nut harvesters also characterized 2017 as an exceptional nadir in production – a sentiment echoed in popular media across the Amazon basin^19^.

The year 2015 was a “Very Strong” El Niño year, which followed immediately on a “Weak” one (2014)^20^. These years relate to our 2017 production because of > 15-month fruit maturation lag times. Such El Niño events yield sunny, dry conditions in our study region. Over the 10-year study, VAP for 2017 production was the highest ranked (26.27 hPa), and 2016 was the second highest (25.37 hPa) during the DTF period; however, precipitation totals reveal that these were the 5th and 9th driest production years (SI Table S2), signaling back-to-back years of persistent high atmospheric moisture but low precipitation. While increases in solar radiation can boost forest productivity^21,22^, persistent dry conditions and higher accompanying temperatures induce tree stress^23^, and ultimately higher mortality^24^. As a canopy emergent, *B. excelsa* crowns are exposed to greater radiation levels and higher evaporative demand. Hence, they are predicted to be particularly sensitive to drought due to hydraulic stress^25^, potentially exacerbated by increased water column tension in such exceptionally tall trees^23^. Still, such large trees access stored groundwater via deep roots more than previously assumed^26^, and fluctuations in water supply can be moderated by internal storage in stems, roots and leaves^27^. It is unknown, however, the extent to which two successive El Niño years may have impacted groundwater recharge and storage, and aggravated overall tree stress. There is evidence that canopy trees are resilient to normal Amazonian dry seasons due to deep roots that access water stored from wet season precipitation^3,28^; yet they are more vulnerable to extended tropical droughts, as demonstrated by the higher rates of large tree, drought-related mortality^29^. Corlett^23^ suggested that this tall tree vulnerability can be attributed to the physiological challenges of transporting water from drying soil through lengthy water conduits to exposed leaves. *B. excelsa* demonstrates drought avoidance by losing leaves during the dry period, but only for a few days in our study region^30^, where deciduousness is unexceptional and average rainfall falls short of ~ 2000 mm expected for evergreen tropical forests^31^. Finally, drought inducement experiments have demonstrated that lower rainfall levels over time negatively affect tropical tree fruit production. Throughfall exclusion over a 4-year period had a cumulative negative effect on fruit production (− 12%) of a sub-canopy tropical Rubiaceae, but differences were only significant in 1 year^32^.

Delayed rainy season onset also may have influenced the extremely low 2017 fruit production. In our region, the rainy season typically begins in September, yet the key 6-month rainfall (DTF; June through November) period that influenced 2017 production was the lowest in our 10-year data set. Moreover, of the entire 117-year CRU data set, the 2017 DTF period was the 16th lowest on record (SI Table S2), indicating that rainy season onset was delayed beyond norms. Since 1979, there has been a delay in dry season end dates (or rainy season onset) and an increase in dry season length for southern Amazonia^33^. Grogan and Schulze^34^ reported that delayed rainy season onset had a negative effect on tropical canopy tree growth, but they did not track fecundity. Finally, negative correlations between fruit production and minimum temperatures during both DPF and DTF (dry season prior to, and through flowering, respectively), particularly in Cachoeira, are consistent with other tropical studies that have showed clear negative effects of high nighttime temperatures on tropical tree growth^22^. In sum, evidence suggests that dry, and perhaps warming, conditions may have produced cascading effects that compromised 2017 fruit production at both sites (Table S2). Still, Cachoeira responded better than Filipinas not only in 2017, but across all years, as indicated by highly significant site effects across models.

Given these results, we explored the role that site differences might play in fruit production*.* Previous studies have detected subtle differences in demographic structures at our sites, indicating the presence of smaller *B. excelsa* individuals in the Filipinas population, but without a clear attribution to ecological or socioeconomic factors^9^. While Cachoeira has a longer history of disturbance (i.e., low-intensity timber harvest), which could influence the dominance of *B. excelsa*, we lack evidence that this disturbance influences production. Despite close proximity, our sites are located in different watersheds, and are characterized by slightly different forest types and soil characteristics. Specifically, Cachoeira’s significantly higher levels of P and K (Table 1) are informative, as soil P has been positively linked to higher levels of *B. excelsa* production^11,17^. Costa^35^ showed that *B. excelsa* can be productive in acidic, less fertile soils, while suggesting that Ca is a key macronutrient for this species.

Site quality has been used extensively to explain and predict productivity across diverse forest types for decades^36^, and inclusion of more site variables (such as depth to water table) would likely yield improved explanations for Cachoeira’s comparatively superior production. Notwithstanding, individual tree differences, regardless of site, offer further fruit production insights. As with almost all trees, *B. excelsa* reproductive status and fruit production levels are explained by DBH^12,16,37–39^, with the most productive trees in the 100–150 cm DBH range^11^. Moreover, DBH for these trees is correlated with crown size^17^, which was a significant and positive explanatory variable for all our production models, although less so for large trees (≥ 100 cm DBH) in Cachoeira versus Filipinas (Table 2, Models 4a & b). Large crowns of individual trees imply greater photosynthetic capacity and sturdy physical structures that support carbohydrate and nutrient demands of the large *B. excelsa* fruits. Large-diameter trees with big crowns produce more fruits. Furthermore, these trees are tall; all exhibit dominant or co-dominant canopy positions, suggesting fairly unlimited access to light. Notably, while basal area growth was a significant predictor of fruit production in trees < 100 cm DBH, it was not in large trees (Table 2, Models 3a-b). This finding corroborates a previous analysis which suggested that once healthy *B. excelsa* individuals attain sizeable girth and maturity, they allocate resources more equitably to both fruit production and basal area increment^17^.

Sapwood area of individual trees also was implicated in fruit production variation in our models, but varied by site and year, and seemed specific to large trees (Table 2). While sapwood area did not explain fruit production in most years, in 2017, Cachoeira trees with large sapwood areas produced significantly more fruit than those with small areas (Models 3b and 4a, Fig. 6). Year 2017 production bore witness to two successive El Niño years and an abnormally delayed rainy season. Further evidence of the positive effect of sapwood on fruit production under dry conditions came from our climate model. Model 2 revealed that higher VAP levels (more moist air) limited fruit production, but this was mitigated when trees had more sapwood area, particularly in Cachoeira (Fig. 4). Sapwood mitigation of drought was also evident in Filipinas in 2013. After 2017, this was the second driest 6-month June–November (DTF) in our data, and in Filipinas, trees with large sapwood areas produced significantly more fruits than those with small. In sum, the more sapwood, the more likely any individual tree could continue producing fruits even in the face of atmospheric conditions.

Sapwood plays a key role in tree-water relations, and it is unsurprising that sapwood area was positively correlated to *B. excelsa* fruit production (r = 0.37), regardless of year or site. When dry conditions were sustained over abnormally long periods, however, sapwood area seemed to be a key trait that differentiated fruit production. In their study of biomass growth of tropical canopy trees, van der Sande et al.^40^ reported that sapwood area strongly explained aboveground biomass growth. While sapwood assures water and nutrient transport from roots, for large trees, sapwood capacity to store water may be most important. Goldstein et al.^41^ reported that canopy trees with larger sapwood areas maintained maximum transpiration rates for a longer fraction of the day, noting that water stored in stem tissues can reach canopy leaves more quickly than soil water, which can take days to ascend to tropical tree crowns.

In both our study sites, favorable climate and site conditions have supported viable *B. excelsa* populations and related fruit production over generational timeframes. Our 10-year data, however, permitted linkage of annually disparate weather conditions with individual *B. excelsa* tree fruit production. Individual tree level research facilitates close-up examination of the biological unit that responds directly to weather and site interactions^40^, illuminating more nuanced understanding of how site-level resources are internally allocated to produce a seed crop in any given year. In both sites, persistent dry conditions (two consecutive low rainfall years) coupled with delayed rainy season onset seemed to have had cumulative negative effects on 2017 fruit production. Given that *B. excelsa* individuals can live for centuries, isolated low fruit production years are unlikely to result in long-term population declines, but they certainly affect short-term harvester incomes. For example, the weather anomaly that seemed key to low 2017 harvests was a concerning event that reverberated throughout Amazonia. Of potentially greater concern, however, is the extent to which long-term climatic changes might modify *B. excelsa* mortality. Using 14 years of data, Bertwell et al.^6^ found no evidence that isolated years of drought threatened population stability, but undoubtedly the possibility of tree mortality increases after multiple years of low rainfall^42^. That fruit production was also linked to soil characteristics opens a door for potential individual tree nutrient manipulation to enhance production. Our study also showed that sapwood area was positively correlated with fruit production, and that in dry years, larger sapwood areas seemed to guard against low fruit production of the largest and presumably most productive trees. Is it possible (and even advisable) to attempt to delay heartwood formation to produce more drought resistant individuals?

On a much larger scale, the importance of maintaining economically viable *B. excelsa* populations on the landscape cannot be overemphasized. Contemporary significance of Brazil nut fruit production to Amazonian economies and conservation of biodiversity and sociocultural values has been highlighted by analysis of estimated Brazil nut rents across the Brazilian Amazon^43^, though much of these Brazil nut-rich forests remain unharvested. Detailed examination of the Amazonian state of Rondônia, for example, reveals that ~ 7.5 million ha (~ 1/3 of the state) are demarcated for sustainable use (i.e., Indigenous Territories, Extractive Reserves)^15^, and if fully exploited, could yield more Brazil nuts than are currently harvested in all of Amazonia. With fairly steady increases in Brazil nut prices over the last decades, Indigenous Amazonians (who occupy 27% of the Brazilian Amazon^44^), increasingly are breaking into Brazil nut markets. While continued monitoring is necessary to ensure sustainable harvests and Brazil nut populations, spatially explicit models that uncover associations between *B. excelsa* distributions and human presence in the Amazon historically, and into the future^16,45^, are encouraging. Furthermore, recent projections suggest that *B. excelsa* and its *Dasyprocta* sp. disperser are resilient to climate change itself, although the co-occurrence of *B. excelsa* and its pollinators was projected to diminish by almost 80% by Year 2090^46^. Significant contemporary threats to Brazil nut sustainability also undoubtedly include felling of reproductively mature trees^6^ and conversion of Brazil nut-rich forests to other uses^47,48^. Disentangling the drivers of fruit production contributes to better scientific understanding of climatic, site-specific and endogenous factors that control fruit and seed production of long-lived, canopy trees. The *B. excelsa* case also highlights factors that sustain this important species on the conservation landscape, while providing potential pathways to increase productivity of extant trees that economically support both local families and a thriving extractive industry.

## Materials and methods

### Study sites

The study was conducted in: Filipinas (− 10.7770°, − 68.6644°) located in Extractive Reserve Chico Mendes, and Chico Mendes Agro-Extractive Settlement Project (informally known as Cachoeira) (− 10.8282°, − 68.3916°)^49^. Annual average rainfall at these two sites is 1770–1880 mm, with a pronounced dry season from June to August when average rainfall is < 50 mm per month^50^. Censuses of all *B. excelsa* individuals ≥ 10 cm DBH revealed statistically similar size distributions (Kruskal–Wallis *P* = 0.84), with population densities of 1.35 and 1.82 trees ha^−1^ in Filipinas^49^ and Cachoeira, respectively. (See Supplemental Information [SI] for further details.) Modeling studies from these two populations reveal that trees in Cachoeira on average reach reproductive maturity (40 cm DBH) far earlier than those of Filipinas (83 versus 167 years)^6^, suggesting that despite smaller diameters, populations in Filipinas could be older.

### Sampled populations and field measurements

From the censuses, a stratified sample of reproductively mature *B. excelsa* adults (50–219 cm DBH) was selected in each site across 10 DBH classes (nine 10-cm DBH classes and one of trees ≥ 150 cm DBH), roughly in proportion to the population. We excluded trees with overlapping crowns or fruitfall areas, and those that died or were affected by road construction or pasture expansion, leaving 129 sample trees well-distributed spatially across each study landscape.

Tree DBH was assessed annually, while other tree attributes were assessed once during the study, including: crown position, area and form, tree location and elevation, competition from neighboring trees, and sapwood area (see SI). Each year during 2010–2015 and 2017–2019, fruit production of each tree was measured after fruitfall in February. While ground counts do not reflect absolute counts of total fruit production due to some removal by scatterhoarding *Dasyprocta* sp., these errors are estimated to be small (~ 5%; L.H.O. Wadt, unpublished data) and thus not considered.

### Supplementary data

Because onsite weather data were not available in our remote study areas, we obtained data from the Climate Research Unit (CRU; version TS v4.03^51^). CRU data are generated on a 0.5° grid, and have been evaluated and applied in studies of vegetation-climate interactions and tropical rainforest climates (e.g.,^52^). Monthly climate data through 2018 were extracted from four grid points closest to Cachoeira and Filipinas (http://wps-web1.ceda.ac.uk/submit/form?proc_id=Subsetter), including: average, minimum, maximum and range of temperature (°C), total precipitation (mm), number of wet days, percentage cloud cover, potential evapotranspiration (PET; mm/day), and vapor pressure (VAP; the partial pressure of water vapor in the atmosphere; hPa). Using linear interpolation, climate variables were estimated for each site; however, site-specific values of all variables were extremely close. Soil data were available from an independent study, collected in two 600 m^2^ plots in each site, yielding measures of key soil nutrients and structural characteristics. Because soil data were not tied to particular trees, only site-level comparisons were possible (see SI for details).

### Statistical analyses

#### Fruiting patterns

Following Kainer et al.^11^, we quantified fruit production variability at the individual level (*CVi*) and population level (*CVp*), and the mean individual fruiting synchrony (*S*). We also tested for bi-annual patterns in production (high years followed by low ones) with the autocorrelation coefficient at a lag of 1 year (AR(1)).

#### Relationship between fruit production and climate

Because the Brazil nut reproductive cycle could be influenced by weather conditions more than two years previous to fruitfall, we initially explored correlative relationships between fruit production and climate variables over various time periods including up to a 3-year lag. Based on these preliminary findings and Brazil nut phenology research and regional seasonal climatic variation, however, we focused on the 3-month dry season, which occurs annually from June–August, as this is a critical period for successful fruit development. We defined the *dry season prior to flowering* (DPF) as the 3-month dry season immediately prior to when Brazil nut in Acre is observed to initiate flowering (L.H.O. Wadt; unpublished data), which occurs ~ 21 months prior to fruitfall (e.g., June–August 2010 DPF relates to fruit production collected in February 2012). We also hypothesized that weather during flowering immediately after this period could play an important role. The *dry season through flowering* (DTF) period was defined to include the June–August dry season and extend through the flowering period ending in November (e.g., for 2012 fruit production, DTF is June–November 2010). We summarized climate variables, summing total precipitation and averaging all other variables during these critical periods of fruit development for each annual production cycle. The Spearman correlation between annual fruit production and each climate variable was computed as a preliminary step in testing hypotheses relating production to climate.

#### Fruit production models

Two types of models were estimated with tree-level variables: one with year, and another with climate variables. Mixed model analyses were conducted, utilizing a repeated measures framework. Because data contained more zeroes than would be expected under a Poisson distribution, we modeled annual fruit production per tree with a Generalized Poisson distribution, which is similar to that of the negative binomial, but includes heavier tails^53^. This model was implemented via the SAS procedure PROC GLIMMIX (SAS/STAT(R), Version 9.4). Random effects were included to account for data taken in multiple years on the same tree. We compared and tested various correlation structures, including compound-symmetric, unstructured, and autoregressive, to obtain optimal model fit.

First, our base model (Model 1) was estimated with predictor variables included for: site, year of fruitfall, DBH, crown size, vine loading, crown position, crown form, and elevation. Interactions between year and site, and the other explanatory variables were also included. Previous analyses have shown a strong correlation between crown size and DBH; thus, models were formulated separately using each of these variables. Second, to test hypotheses about climate, year was dropped, and Model 2 included explanatory variables for climate over the corresponding DPF and DTF periods. We standardized all predictors prior to model fitting, and utilized a modified backwards-stepwise model selection method, dropping non-significant variables and monitoring the AIC and Generalized Chi-square/DF statistic. Variables were eliminated from the model unless their removal resulted in an increase in the AIC, or a divergence of the Chi-square/DF statistic from 1.0. Where effects were significant (*P* < 0.05), marginal means were generated, holding all other variables in the model at their average values. Comparisons among effects levels were made via the Tukey-test. Where interactive effects were significant, tests of simple effects^54^ were applied to test each individual effect.

To further disentangle drivers, models were then estimated with data subsetted in two ways. Because trade-offs between growth and production are different for smaller versus larger *B. excelsa* reproductive adults^17^, we first compared models for small (< 100 cm) versus large (≥ 100 cm DBH) trees (Models 3a and 3b). We then compared models by site, splitting data for Cachoeira versus Filipinas (Models 4a and 4b). All tree-level variables were included along with interactions with year as above, using the same modeling and model selection criteria.

## Supplementary Information

Supplementary Information.
