# Author Correction: Comparative models disentangle drivers of fruit production variability of an economically and ecologically important long-lived Amazonian tree

**DOI:** 10.1038/s41598-023-31310-7

**Published:** 2023-03-21

**Authors:** Christina L. Staudhammer, Lúcia Helena O. Wadt, Karen A. Kainer, Thiago Augusto da Cunha

**Affiliations:** 1 Department of Biological Sciences, University of Alabama, P.O. Box 870344, Tuscaloosa, AL 35487 USA; 2 Centro de Pesquisa Agroflorestal de Rondônia (Embrapa Rondônia), BR 364, km 5,5, Caixa Postal 127, Porto Velho, Rondônia CEP 76815-800 Brazil; 3 School of Forest Resources and Conservation, and Center for Latin American Studies, University of Florida, P.O. Box 110410, Gainesville, FL 32611-0410 USA; 4 Universidade Federal do Acre, Rodovia BR 364, Km 04, Distrito Industrial, Rio Branco, Acre Brazil

Correction to: *Scientific Reports* 10.1038/s41598-021-81948-4, published online 28 January 2021

The original version of this Article contained errors in the Discussion, in its interpretation of the effect of vapor pressure (VAP).

Consequently, in the Discussion section,

“Furthermore, the model also indicated that when drier atmospheric conditions (represented by VAP) were present and extended beyond the dry season into the flowering period, fruit production tended to be reduced.”

now reads:

“Furthermore, the model also indicated that when wetter atmospheric conditions (represented by VAP) were present and extended beyond the dry season into the flowering period, fruit production tended to be reduced.”

“Over the 10-year study, VAP for 2017 production was the lowest ranked (26.27 hPa), and 2016 was the second lowest (25.37 hPa) (SI Table S2), signaling back-to-back years of persistent low atmospheric moisture.”

now reads:

“Over the 10-year study, VAP for 2017 production was the highest ranked (26.27 hPa), and 2016 was the second highest (25.37 hPa) during the DTF period; however, precipitation totals reveal that these were the 5th and 9th driest production years (SI Table S2), signaling back-to-back years of persistent high atmospheric moisture but low precipitation.”

“Model 2 revealed that higher VAP levels (drier air) limited fruit production, but this was mitigated when trees had more sapwood area, particularly in Cachoeira (Fig. 4). Sapwood mitigation of drought was also evident in Filipinas in 2013. After 2017, this was the second driest 6-month June–November (DTF) in our data, and in Filipinas, trees with large sapwood areas produced significantly more fruits than those with small. In sum, the more sapwood, the more likely any individual tree could continue producing fruits even in the face of a drier atmosphere.”

now reads:

“Model 2 revealed that higher VAP levels (more moist air) limited fruit production, but this was mitigated when trees had more sapwood area, particularly in Cachoeira (Fig. 4). Sapwood mitigation of drought was also evident in Filipinas in 2013. After 2017, this was the second driest 6-month June–November (DTF) in our data, and in Filipinas, trees with large sapwood areas produced significantly more fruits than those with small. In sum, the more sapwood, the more likely any individual tree could continue producing fruits even in the face of atmospheric conditions.”

The original Article has been corrected.

