# Supplementary material for: Comparative models disentangle drivers of fruit production variability of an economically and ecologically important long-lived Amazonian tree
Source: Sci Rep. 2021 Jan 28;11:2563. doi: 10.1038/s41598-021-81948-4 (PMC7843625; doi:10.1038/s41598-021-81948-4)
Supplement: Supplementary file 1 — Supplementary Information. [file 41598_2021_81948_MOESM1_ESM.pdf]

Supplemental Information

Comparative models disentangle drivers of fruit production variability of an economically and ecologically important long-lived Amazonian tree

Staudhammer, Christina L., Wadt, Lúcia H.O., Kainer, Karen A., da Cunha, Thiago Augusto

Study sites

The Filipinas study site was located in one unlogged 420-ha extractivist landholding in the southeastern portion of Extractive Reserve Chico Mendes (Colocação Rio de Janeiro in Seringal Filipinas). The Reserve maintains 92% forest cover [1]. Filipinas is dominated by open forest with bamboo and/or palms, with a small area classified as dense forest [2]. A 2003 forest inventory in the study area estimated that tree density was 400 individuals ha^{-1}, with 20.5 m^2 ha^{-1} of basal area [3]. A 2001-02 inventory of all B. excelsa individuals ≥ 10 cm diameter at breast height (DBH; measured at 1.3 meters above ground level) revealed a population density of 1.35 trees ha^{-1} and an average DBH of 86.1 ± 45.0 cm [4]. Of 145 trees < 50 cm DBH inventoried, only 20% had initiated fruit production, while 96% of 404 trees ≥ 50 cm DBH were reproductive [4]. Of this latter subset, almost all had dominant or co-dominant crown positions.

Our second study site was located in the Chico Mendes Agro-Extractive Settlement Project (informally known as Cachoeira), which maintains 90% forest cover. Since 2000, Cachoeira has had small-scale timber extraction certified by the Forest Stewardship Council, and in part due to its greater socioeconomic activity, Cachoeira has a relatively greater number of trails [3]. This site shares similar forest types as Filipinas, but in different proportions. Cachoeira is dominated by dense forest with a smaller area classified as open forest with bamboo and/or palms [2]. The 2003 forest inventory of the study area in Cachoeira revealed slightly lower tree density but higher basal area in Cachoeira versus Filipinas: 367 trees ha^{-1} and 27 m^2 ha^{-1} of basal area [5]. Our ~200 ha study site at Cachoeira includes parts of three settlement landholdings (see [4] for a map). A 2009 inventory of all B. excelsa individuals ≥ 10 cm DBH revealed a B. excelsa population density of 1.82 trees ha^{-1} and an average diameter of 86.9 ± 44.9 cm. Like Filipinas, the 275 trees > 50 cm DBH inventoried were almost all in dominant or co-dominant crown positions.

A Kruskal-Wallis rank sum test to compare the diameter distributions of the Brazil nut populations (>10 cm dbh in our two study sites revealed that the two sites did not significantly differ (P=0.46; Figure S1). However, previous studies have concluded that Filipinas seemed to have younger Brazil nut stands, with smaller and less numerous adults and more seedlings and saplings [5].

Field and laboratory sampling methods

The same sampling protocols were followed in both locations. Tree DBH was assessed annually. Three crown attributes of each sample tree were assessed once during the study: (1) Crown position was categorized as (a) Dominant (full overhead and side light), (b) Co-dominant (full overhead light), and (c) Intermediate (some overhead or side light) or (d) Suppressed (no direct light); (2) Crown radii were measured in four cardinal directions for trees with regular crowns, and with 8 cardinal directions for trees with irregular crowns; and (3) Crown form was categorized as: (a) Good, (b) Tolerable, or (c) Poor. Crown size was estimated as the area from 4 or 8 radii, using an ellipsoid formula. Using a high-accuracy geodesic GPS unit, tree location and elevation (previously implicated as an explanatory variable in harvester estimates of *B. excelsa* fruit production [6]) were also noted. Competition from neighboring trees was evaluated in 2018. We used the Bitterlich method, whereby competing trees are selected through evaluation of their size and distance from each subject (*B. excelsa*) tree. Using a prism with a 2.3 m2/ha basal area factor, we measured the DBH of all competing trees. For each tree, we calculated the basal area

and number of competitors in total and in three DBH classes: >30 cm, >40 cm, and those larger than the subject tree.

In 2010, sapwood data were collected using a standard 5-mm increment borer at 1.3 m height, extracting two perpendicular cores ~150mm in length per tree. Each core was visually cross-dated [7] at the Forest Science Laboratory on the Universidade Federal do Acre campus, and annual ring widths on each core were measured to an accuracy of 0.01 mm using a Lintab linear table (Frank Rinn S.A., Heidelberg, Germany) and TSAP-Win tree ring software [8]. To better visualize the macroscopic anatomical structure of the wood, we used a sharpened knife to cross cut the cores rather than polish with sandpaper. Annual ring width on each tree was estimated as the average of the two core measurements taken on each tree, by year. The length of sapwood contained in each increment core was easily delimited by identifying regions of open porous cells and the contrasting wood color of the heartwood. The sapwood area was calculated using the tree DBH and the average sapwood length measured from the cores, assuming a circular bole.

Soil data were available from a separate study conducted at the two sites. Two 600 m^2 plots in each were delineated and soil data were collected using a 100 x 60 m grid. Sixty soil samples of 300 g at each grid intersection were collected at two depths: 0-20 cm for nutrient content and soil texture, and 7-13 cm for density and porosity measures. Samples were dried, sifted and analyzed using standard methods at the Soil and Plant Analysis Laboratory of Embrapa-Western Amazonia [9]. Available Ca^{2+}, Mg^{2+}, K^{+}, Al^{3+} and P contents, pH, potential acidity ($Al^{3++}H+$), total N, organic carbon and clay content were measured on the 0-20cm samples via the pipette method. Soil porosity (SP) was calculated based on soil bulk (SBD) and particle (PD) density (measured using the rubber balloon method), as: SP = (1 – SBD/PD).

References

1. SEMA (Secretaria de Meio Ambiente). *Diagnostico socioecon omico e cadastro da Reserva Extrativista Chico Mendes – Plano Resex sustentavel*. (Secretaria de Estado de Meio Ambiente do Governo do Estado do Acre - ICMBIO, 2010).

2. Zoneamento Ecologico-Econômico do Acre (ZEE), F. I. *Documento Sintese – Escala 1:250.000*. (2010).

3. Wadt, L. H. O., Kainer, K. A., Staudhammer, C. L. & Serrano, R. O. P. Sustainable forest use in Brazilian extractive reserves: Natural regeneration of Brazil nut in exploited populations. *Biol. Conserv.* **141**, 332–346 (2008).

4. Wadt, L. H. O., Kainer, K. A. & Gomes-Silva, D. A. P. Population structure and nut yield of a Bertholletia excelsa stand in Southwestern Amazonia. *For. Ecol. Manage.* **211**, 371–384 (2005).

5. Wadt, L. H. O., Kainer, K. A., Staudhammer, C. L. & Serrano, R. O. P. Sustainable forest use in Brazilian extractive reserves: Natural regeneration of Brazil nut in exploited populations. *Biol. Conserv.* **141**, (2008).

6. Thomas, E. *et al.* NTFP harvesters as citizen scientists: Validating traditional and crowdsourced knowledge on seed production of Brazil nut trees in the Peruvian Amazon. *PLoS One* **12**, e0183743 (2017).

7. Stokes, M. A. & Smiley, T. L. *An introduction to tree-ring dating*. *An introduction to tree-ring dating* (University of Arizona Press, Tucson, 1996).

8. Rinn, F. TSAP-Win, Software for tree-ring measurement, analysis and presentation. (2003).

9. Embrapa Solos. *Manual de métodos de análise de solo*. (Embrapa Solos, 2017).

Supplementary Tables and Figures

Table S1. Type 3 tests of fixed effects from generalized Poisson mixed models of annual Brazil nut production, using (1) year as the indicator of annual conditions, and (2) climate variables instead of year, (3a) large trees (≥ 100 cm), (3b) small trees (< 100 cm DBH), (4a) large trees in Cachoeira, and (4b) large trees in Filipinas. Models (3)-(5) use year as the indicator of annual conditions. VAP=vapor pressure (hPa) during the dry season through flowering (DTF), Wet = number of wet days during the dry season prior to flowering (DPF). (Num DF = numerator degrees of freedom, Den DF = denominator degrees of freedom).

Model	Effect	Num DF	Den DF	F Value	Pr > F
(1) Year	Year	8	1968	64.26	<.0001
	Site	1	244	104.84	<.0001
	Year x Site	8	1968	39.32	<.0001
	Crown Size	1	1968	26.53	<.0001
	Crown Form	2	1968	8.62	0.0002
	Elevation	1	1968	0.64	0.4254
	Elevation x Year	8	1968	2.25	0.0216
	Sapwood area	1	1968	0.05	0.8153
	Sapwood area x Year	8	1968	3.57	0.0004
(2) Climate	VAP (DTF)	1	2018	248.6	<.0001
	Site	1	249	115.47	<.0001
	Site x VAP (DTF)	1	2018	25.12	<.0001
	Sapwood area	1	2018	0.01	0.9271
	Sapwood x VAP (DTF)	1	2018	5.03	0.025
	Wet (DPF)	1	2018	30.91	<.0001
	Crown Size	1	2018	26.84	<.0001
	Crown Form	2	2018	8.6	0.0002
(3a) DBH < 100 cm	Year	7	784	22.07	<.0001
	Site	1	114	104.97	<.0001
	Year x Site	7	784	16.98	<.0001
	Crown size	1	784	19.49	<.0001
	Crown form	2	784	6.69	0.0013
	Elevation	1	784	0.34	0.5624
	Elevation x Year	7	784	3.07	0.0034
	Elevation x Site	1	784	0.14	0.7083
	Elevation x Site x Year	7	784	2.36	0.0216
	Sapwood area	1	784	3.14	0.077
	BA growth	1	784	13.71	0.0002
	BA growth x Year	7	784	2.36	0.0219
	BA growth x Site	1	784	7.78	0.0054
(3b) Trees ≥ 100 cm	Year	8	1013	36.46	<.0001
	Site	1	129	36.13	<.0001
	Year x Site	8	1013	16.34	<.0001
	Crown size	1	1013	15.98	<.0001
	Elevation	1	1013	4.08	0.0438
	Sapwood area x Year	1	1013	0	0.9894
	Sapwood area x Site	8	1013	1.77	0.0799
	Sapwood area x Year x Site	1	1013	0	0.9739
	Sapwood area	8	1013	2.9	0.0034
(4a) Cachoeira	Year	8	498	10.18	<.0001
	Crown size	1	498	6.02	0.0145
	Sapwood area	1	498	0.11	0.7349
	Sapwood area x Year	8	498	4.8	<.0001
(4b) Filipinas	Year	8	515	40.32	<.0001

Crown size	1	515	8.83	0.0031
Sapwood area	1	515	0.02	0.8771
Sapwood area x Year	8	515	2.05	0.0388
Elevation	1	515	1.51	0.2197

Table S2. Climate variables by production year summarized by development period (DPF = dry season prior to flowering, DTF = dry season through flowering).

Variable	Time Period	2010	2011	2012	2013	2014	2015	2016	2017	2018	2019
Cumulative Precipitation (mm)	DPF	79.1	152.9	51.4	17.4	66.0	170.8	128.8	102.9	97.6	62.1
	DTF	500.4	716.2	490.4	305.7	582.6	574.7	533.1	388.1	534.1	403.5
Average Temperature (˚C)	DPF	24.6	24.5	24.7	25.1	24.9	24.6	25.1	26.0	25.2	25.1
	DTF	25.5	25.7	25.8	26.1	26.1	25.5	26.2	26.9	26.2	25.9
Average minimum temperature (˚C)	DPF	17.6	18.5	18.0	18.6	18.5	18.4	18.9	19.5	18.2	18.6
	DTF	19.1	19.9	19.5	19.8	20.1	19.8	20.1	20.8	19.8	19.9
Average maximum temperature (˚C)	DPF	31.6	30.5	31.6	31.7	31.3	30.7	31.4	32.5	32.3	31.6
	DTF	32.0	31.6	32.2	32.4	32.2	31.3	32.3	33.0	32.6	32.0
Average temperature range (˚C)	DPF	14.0	12.0	13.5	13.1	12.8	12.3	12.5	12.9	14.0	13.0
	DTF	12.9	11.7	12.7	12.6	12.1	11.5	12.2	12.1	12.8	12.1
Average cloud cover (%)	DPF	51.5	63.9	54.8	56.8	59.5	62.7	60.4	58.0	52.2	56.9
	DTF	61.0	68.8	62.6	63.3	66.8	70.0	64.9	65.3	62.2	63.7
Average number of wet days	DPF	4.2	7.9	4.2	4.7	7.6	7.1	7.9	6.4	4.1	3.8
	DTF	8.1	11.4	9.0	9.3	11.4	12.6	10.6	11.0	8.4	9.5
Average VAP (hPa)	DPF	22.6	23.3	23.0	23.7	23.5	23.5	23.9	24.5	23.3	23.2
	DTF	24.8	25.0	24.7	25.2	25.3	25.2	25.4	26.3	25.2	25.0

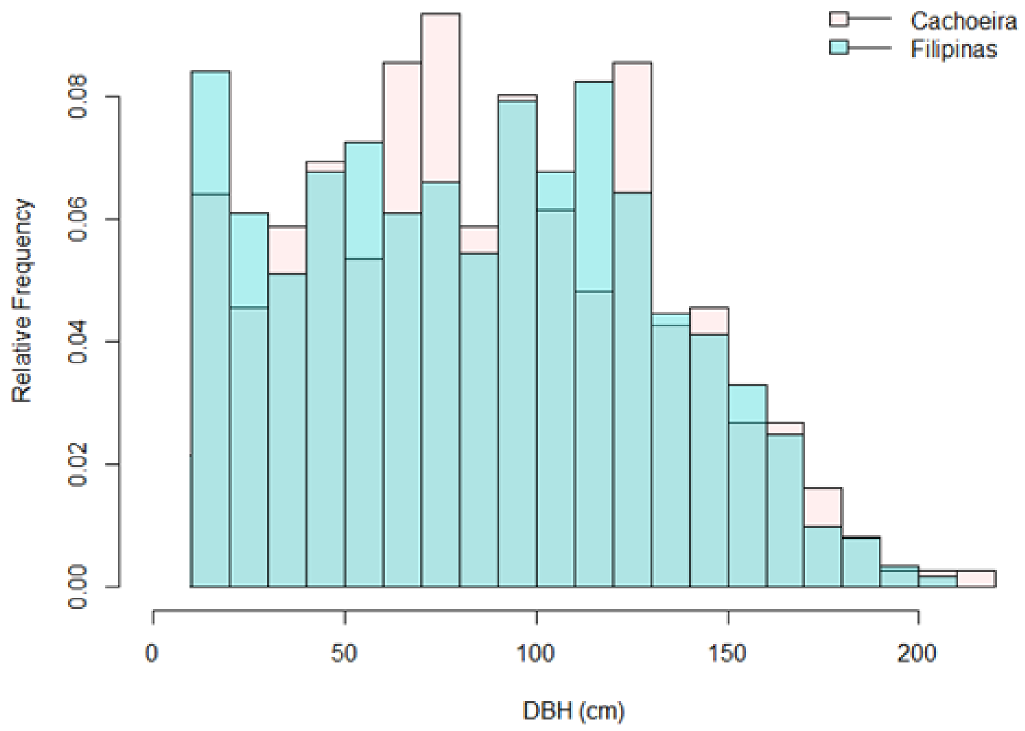

Figure S1. Comparison of diameter distributions from censuses of all Brazil nut individuals >10 cm DBH in each of the two study sites. DBH=diameter at breast height.
